# Over Fifty Years of Life, Death, and Cannibalism: A Historical Recollection of Apoptosis and Autophagy

**DOI:** 10.3390/ijms222212466

**Published:** 2021-11-18

**Authors:** Mahmoud Izadi, Tayyiba Akbar Ali, Ehsan Pourkarimi

**Affiliations:** Division of Genomics and Translational Medicine, College of Health and Life Sciences, Hamad Bin Khalifa University, Doha 34110, Qatar; maiz30979@hbku.edu.qa (M.I.); TAkbar@hbku.edu.qa (T.A.A.)

**Keywords:** apoptosis, autophagy, *C. elegans*, caspase, Bcl-2, Apaf1, electron microscopy

## Abstract

Research in biomedical sciences has changed dramatically over the past fifty years. There is no doubt that the discovery of apoptosis and autophagy as two highly synchronized and regulated mechanisms in cellular homeostasis are among the most important discoveries in these decades. Along with the advancement in molecular biology, identifying the genetic players in apoptosis and autophagy has shed light on our understanding of their function in physiological and pathological conditions. In this review, we first describe the history of key discoveries in apoptosis with a molecular insight and continue with apoptosis pathways and their regulation. We touch upon the role of apoptosis in human health and its malfunction in several diseases. We discuss the path to the morphological and molecular discovery of autophagy. Moreover, we dive deep into the precise regulation of autophagy and recent findings from basic research to clinical applications of autophagy modulation in human health and illnesses and the available therapies for many diseases caused by impaired autophagy. We conclude with the exciting crosstalk between apoptosis and autophagy, from the early discoveries to recent findings.

## 1. Brief History of Apoptosis

The word “apoptosis” or “falling leaf”, perhaps one of the most cited scientific terms in literature, was not chosen by a geneticist or a biochemist but by a linguist at the University of Aberdeen named Cormack [1]. The term apoptosis, often used synonymously with programmed cell death (PCD), was first introduced to scientific literature in 1972 [1,2]. After Kerr et al. coined the term apoptosis and described its cytological features, the field exploded. However, the use of the term dates back to ancient Greece. Although unrelated to programmed cell death, the first use of apoptosis was by Hippocrates (~460–370 BC) in his book *Mochlicon* while describing tissue decomposition. The word was later used by Galen (129–201 AD) in the context of wound healing to describe the falling of scabs [3,4,5]. Karl Vogt demonstrated the first report of cell death in the notochords and nearby cartilages of metamorphic toads in 1842 [6].

In 1858, a German pathologist, Rudolph Virchow, defined two distinct types of cell death: necrosis and necrobiosis. It took more than a century to show that the described morphological features of necrobiosis are in line with what we now know as programmed cell death [7]. However, detailed morphological features and mechanisms were not apparent at the time. The only morphologically well-established mechanism of cell death was a passive, non-controlled cell death referred to as coagulative necrosis, which was introduced in 1889 by Julius Cohnheim and known to result from the irreversible disturbance of cell homeostasis, such as autolysis [6,8,9]. Flemming, in 1885, reported the canonical feature of apoptotic death, the DNA condensation or pyknosis (from Greek “pyknono”, meaning “to condense”) in the epithelial cells of ovarian follicles [10]. Furthermore, he described that the nuclei containing the condensed DNA would eventually break down and disappear, a process he named chromatolysis because of the disintegrated chromatin in the cells undergoing death as a consequence of mechanical and chemical stimuli [6]. Another type of cell death was gradually detected in untreated malignant tumors, relapsed tumors, and even in normal tissues that were active and controlled intrinsically [11,12,13,14]. Several reports suggested that this cell death was involved in organ development and atrophy [14,15,16]. Based on its morphological features, this type of cell death was known as shrinkage necrosis and could be triggered by physiological or pathological stimuli [14,17,18]. These early observations collectively contributed to the birth of one of the fascinating types of death, apoptosis.

Intrinsic cell suicide, defined by Kerr et al. as the controlled cell death triggered in pathological and physiological conditions, was initially discovered as part of a normal developmental process in animals, counterbalancing mitosis [1]. Using electron microscopy, they described cell structural changes that occur during apoptotic death. Unlike cells undergoing necrosis, cells undergoing controlled death do not burst and cause no collateral damage to the neighboring cells and tissues. Hence, controlled cell death does not trigger an inflammatory response characteristic of necrotic cell death [19].

**Figure 1:**
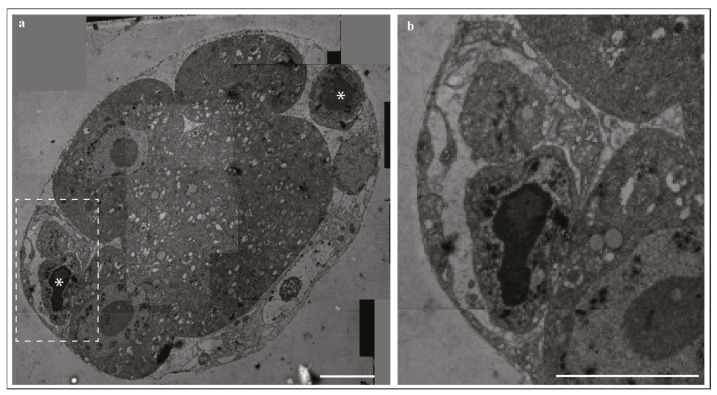
Dying *C. elegans* germ cells show ultrastructure characterization of apoptotic cells. (**a**) Cross-section of the isolated *C. elegans* germline, 24 h after gamma radiation. Two cells undergoing apoptosis (marked with asterisks) exhibit the typical feature of apoptosis and are engulfed with the neighboring cell. The chromatin is highly condensed, and the nucleus is cellularized. (**b**) Magnification of the box shows the complete engulfment of the corpse. Scale bar: 5 μm (Image courtesy of Ehsan Pourkarimi and Anton Gartner, Center for Gene Regulation and Expression, University of Dundee).

Apoptotic bodies, which are spherical cytoplasmic compartments of various sizes and contents, are the main histological feature of apoptotic cells, previously described as councilman-like bodies in the liver [11]. Using an electron microscope, two steps in the morphological alteration of apoptosis were observed: the formation of apoptotic bodies, followed by their engulfment and degradation (Figure 1). These steps were common in various tissues, conditions, organisms, and even developmental stages, including but not limited to embryonic mesenchyme, normal neonatal rat adrenal, human and animal tumors, and liver and adrenal injuries [13,20,21,22,23]. Kerr et al. further characterized the condensation of nucleus and cytoplasm, DNA fragmentation, bleb formation, and the release of apoptotic bodies, which later fuse with lysosomes to digest and recycle the remnants of dead cells [1].

In the 1970s, in a bid to understand how tumor viruses such as SV40 contribute to the carcinogenesis of normal cells, several researchers, including David Lane and Arnold Levine, independently discovered a protein with a mass of 53 kDa (p53) in transformed tumor cells [24,25,26,27,28]. Identifying p53 in virally transformed cancer cells by research groups led by Levine, Lane, and Baltimore introduced this protein as a cellular tumor antigen [25,26,29]. A 1979 PNAS paper published by DeLeo et al. at Memorial Sloan Kettering Cancer Center in New York massively contributed to understanding the mysterious 53 kDa protein [30]. Using chemically induced mouse sarcomas and SV40-transformed cell lines, they re-identified the 53 kDa protein and named it p53. It was evident from DeLeo’s work that p53 expression indeed has a cellular origin. For almost a decade, p53 was considered an oncogene until further research showed that it is mutated or deleted in different cancers, consequently leading to the notion that p53 is indeed a tumor suppressor gene [31,32,33,34]. Since its independent identification by different laboratories, it had been designated several names until the first p53 workshop in the UK in 1983, where the name “p53”, rooted in its supposed molecular mass of 53 kDa, won the battle; this was before it became evident that the molecular mass of human p53 protein is 43.7 kDa [31].

**Figure 2:**
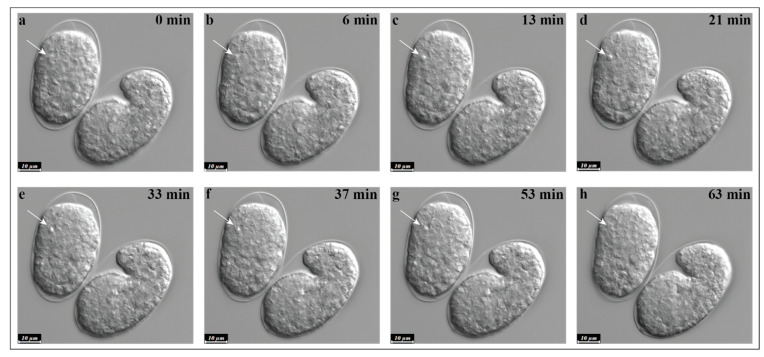
Apoptosis in *C. elegans* embryo. Different stages of developmental apoptosis in *C. elegans* embryo. (**a**) Pre-apoptotic cell; (**b**) apoptosis initiation; (**c**) initiation of engulfment; (**d**–**f**) engulfment; (**g**,**h**) disappearance. Relative time is indicated at the upper right of each frame; Arrow indicates the dying cell at different stages. Scale bar: 10 μm. For the experimental procedure, see the Materials and Methods section.

Using a differential interference contrast (DIC) microscope, which gives depth and contrast to transparent specimens, Sulston and Horvitz directly observed the embryonic and post-embryonic development of the nematode *Caenorhabditis elegans* (*C. elegans*). Sulston and, later, Bob Horvitz identified the precise developmental map of *C. elegans*, a journey from fertilized zygote to an adult worm, and mapped the first developmental lineage of a multicellular organism [35,36]. While analyzing the invariant *C. elegans* lineage, they made an intriguing observation of temporally and developmentally regulated “programmed” cell death. It was evident that at specific developmental timing and based on certain developmental requirements, a number of cells died and disappeared surprisingly quickly. They interpreted PCD as a series of morphological changes, especially in the cell nucleus, including bleb formation, refractivity increment, and, finally, cellular shrinkage and disappearance [15,35]. The initiation and execution of apoptosis occur in three main steps: Cells have to decide to undergo programmed cell death, the execution of apoptosis has to be regulated, and, finally, cells undergoing apoptosis have to be marked and eliminated by phagocytosis (Figure 2, and Video S1).

In 1986, in search of apoptosis-related mutants, Ellis and Horvitz performed a series of excellent genetic screens uncovering the core apoptosis pathway. The identification of apoptosis-defective mutants was aided by discovering mutants with corpse engulfment defects [37,38]. In engulfment-defective mutants, apoptotic corpses persist for much longer compared to the wild-type. This excessive number of morphologically distinct corpses led to the isolation of apoptosis-defective mutants and the discovery of the core apoptotic machinery. Furthermore, the forward genetic screens led to the identification of three complementation groups, viz., cell death abnormal-3 (*ced-3*), *ced-4*, and *ced-9* [37,39,40]. The first two mutants were recessive, while the last behaved as a dominant mutation, suggesting its gain-of-function nature. In a strong *ced-3* and *ced-4* loss-of-function background, the death of the 131 cells that are normally destined to die is compromised. These early observations and, importantly, the isolation of the apoptosis-defective mutants were a turning point in the field for several reasons, viz., the identification of apoptosis-defective mutants confirmed the initial hypothesis of Kerr et al.; apoptosis is indeed an active process and subjected to genetic regulation. The viability of *C. elegans* apoptosis-defective mutants was instrumental in uncovering the different modalities of programmed cell death. Our knowledge of the genetic regulation of apoptosis in *C. elegans* became a cornerstone for understanding apoptosis in the mammalian system, in which apoptosis is linked to cancer and other human diseases, including those involving autoimmunity and neurodegeneration. The importance of early genetic studies in *C. elegans* was further accentuated when the corresponding mammalian genes were cloned and identified.

Around the same time, t 14;18 chromosomal translocation was shown to be the molecular hallmark of follicular lymphoma, one of the most frequently diagnosed blood cancers [41]. The breaking point of such translocation was believed to be upstream of the Bcl-2 (B-cell lymphoma 2) gene, causing the overexpression of Bcl-2 under the regulation of the immunoglobin heavy chain locus, which clearly demonstrates that this overexpression is strongly associated with follicular lymphoma [42,43]. The oncogenic nature of Bcl-2 translocation made scientists believe that Bcl-2 behaves as an oncogene, a fallacy that David Vaux corrected. Overexpressing Bcl-2 cDNA failed to transform various cell lines, such as myeloid and fibroblast cells; in addition, a series of independent studies indicated that Bcl-2 is indeed not an oncogene [44,45,46]. David Vaux made an unexpected observation: while Bcl2-transfected cells failed to generate colonies, they did not die even several days after plating them in growth-factor-free media [44]. This strongly suggested the pro-survival function of Bcl-2, making the puzzle more complicated [44]. Several excellent experiments analyzing Bcl-2-expressing transgenic mice also confirmed the cell-survival-promoting effect of mammalian Bcl-2. Expression of Bcl-2 in mice resulted in an enlarged spleen, yet it failed to promote malignancy [47]. In 1992, Hengartner, Ellis, and Horvitz isolated a dominant *C. elegans* mutation with an apoptosis inhibitory effect, named *ced-9*. This was indeed the first antiapoptotic gene to be isolated in any model system. Hengartner and Horvitz immediately performed a suppressor screen on the isolated antiapoptotic *ced-9*, identifying a loss-of-function mutant of *ced-9*. Inactivation of *ced-9* resulted in excessive apoptosis, with apoptosis occurring in cells that normally were destined to survive [40]. Genetic epistasis placed the antiapoptotic *ced-9* upstream of *ced-3* and *ced-4* [40]. Interestingly, mammalian Bcl-2 expression in worms rescued two-thirds of cells that normally would undergo apoptosis execution, indicating that human Bcl-2 interacts with the *C. elegans* apoptotic machinery [48]. The breakthrough came after sequencing the *ced-9* reading frame; CED-9 surprisingly turned out to be the counterpart of mammalian Bcl-2, a discovery that clearly explained the pro-survival phenotype associated with the overexpression of mammalian Bcl-2 [40]. All in all, these early studies showed how diminished cell death contributes to cancer formation.

In 1992, the sequence of *C. elegans ced-3* was clear to encode a protease related to the caspase family of proteins, and it was evident that the protease activity of CED-3 was the initiator of apoptosis execution [49]. As expected, the mammalian ortholog of CED-3, interleukin-1 beta (IL-1β) converting enzyme (ICE), mimicked the *ced-3* cell death phenotype. Overexpression of both ICE and *C. elegans ced-3* in Rat-1 cells induced apoptosis. [50]. Subsequently, inactivating the active site of mICE by replacing cytosine residue with glycine inhibited its proapoptotic function [50]. Later studies revealed the structure of mICE and confirmed its function as a cysteine protease, later named cysteine-aspartic protease -1 (*caspase-1*) [51,52,53,54,55,56]. Kumar et al. found a series of genes in mice (*Nedd1* to *Nedd10*) and reported that a protein coded by the *Nedd2* gene was similar to ICE and CED-3 and had a high expression in cells undergoing apoptosis during mouse development [57]. Further research identified more proteins related to ICE in the mammalian system and inferred that there is a family of CED-3/ICE cysteine proteases with different substrate specificity and proposed caspase-3 (Yama, Apopain, or 32-kDa putative cysteine protease CPP32) as the mammalian ortholog of CED-3 [53,58,59,60,61,62,63,64,65,66,67].

In 1993, Boise et al. reported the isolation of a novel *bcl-2*-related gene, *bcl-x*, as a regulator of apoptosis [68]. This finding initiated a race to identify other pro- and antiapoptotic Bcl-2-related proteins. Bcl-2 protein family members that are structurally related contain Bcl-2 homology (BH) domains and are characterized in three different groups according to their cell death function: (1) antiapoptotic Bcl-2 members (Bcl-2, Bcl-x, Bcl-W, and Mcl-1) [48,68,69,70], (2) proapoptotic Bcl-2 members (BAK and BAX) [71,72,73,74], and (3) proapoptotic BH3-only domain proteins (NOXA, PUMA, BAD, BIK, BIM, harakiri, and Beclin-1) [75,76,77,78,79,80,81,82,83]. Bcl-2 family members form homodimer and heterodimer protein complexes, and it is the interaction of the antagonistic members that contributes to the life and death decisions of cells. Later, Hengartner and Horvitz characterized the *ced-9* gene in *C. elegans* and reported that this gene encodes a functional ortholog of mammalian *bcl-2*; they also showed the conserved molecular mechanism of apoptosis from nematodes to mammals, a puzzle that was gradually being solved [84,85]. However, the mammalian ortholog of *ced-4* was not discovered by that time.

**Figure 3:**
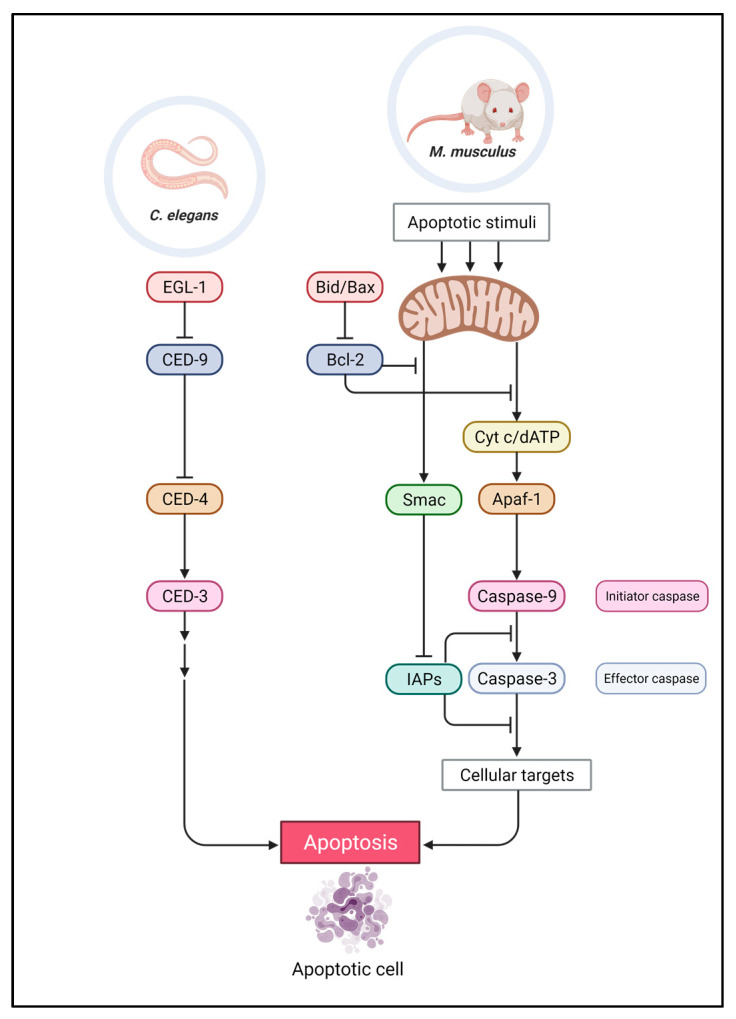
Comparison of apoptosis genetic pathway in *C. elegans* and mammals. The main genes of the core apoptotic machinery of *C. elegans* and mice are demonstrated. The illustration is created with BioRender.com.

In 1996, Liu et al. established a neat cell-free system to assess apoptosis in vitro. They tested protein fractions isolated from Hela cells for the activation of caspase 3 in vitro. Two of the protein fractions were able to induce caspase activity in the cell-free system, suggesting that they consisted of apoptosis-inducing factors, and the fractions were named apoptotic protease activating factor-1 (Apaf-1) and 2 (Apaf-2). Further analysis of the Apaf-2-containing fraction led to isolating a 15 kDa protein band that retained the caspase 3 activation property. Interestingly, the 15 kDa purified protein had a unique pink color with an absorbance peak at 415, 520, and 549 nm, resembling the spectrum of cytochrome c that had been earlier identified. Further peptide sequencing and biochemical analysis of Apaf-2 confirmed that the 15 kDa protein was indeed the mitochondrial cytochrome c [86]. This was the first report to show the involvement of an organelle, the mitochondria, in apoptosis, and it suggested that the release of cytochrome c might be a key step in apoptosis induction, which massively contributed to our understanding of the apoptosis pathway. Moreover, mitochondrial contribution to apoptosis was further confirmed by showing that Bcl-2, in mammalian and *C. elegans* systems, is located on the mitochondrial outer membrane (MOM) and inhibits caspase 3 activation by inhibiting the release of cytochrome c [86,87,88,89,90]. Later, it was shown that the proapoptotic Apaf-1, containing a cytosolic fraction, holds a 130 kDa protein with sequence similarity to that of *C. elegans* CED-4 [86,87]. Shaham and Horvitz in 1996 proposed a genetic pathway for apoptosis in *C. elegans*, which put the function of *ced-4* upstream of or in parallel to *ced-3*, whereas *ced-9* was found to act as a negative regulator of *ced-4* in its upstream (Figure 3). Moreover, they showed that alternative splicing resulted in two *ced-4* transcripts, namely, *ced-4L* and *ced-4S*, which have opposing activities. The former, the more abundant transcript, was previously reported to induce PCD, whereas the latter inhibits cell death [91,92].

Xue and Horvitz, in 1997, further elaborated on the protective roles of CED-9 in apoptosis by indirect inhibition of the proapoptotic protein CED-3 through different mechanisms [93]. In 1997, Spector et al. proposed that a physical interaction between CED-9 and CED-4 controls apoptosis in *C. elegans* [94]. Conradt and Horvitz, in 1998, reported a negative regulator of CED-9 protein coded by the *C. elegans egl-1* (egg-laying-defective) gene that, when overexpressed, led to apoptosis. They analyzed the EGL-1 protein sequence and considered it a BH3-only domain protein that activated PCD through physical interaction with CED-9 and inhibited its interaction with CED-4 [95]. Further investigations of *bcl-2/ced-9* showed their tumorigenic effects and exposed them as targets of anticancer therapy [96]. Horvitz, Sulston, and Brenner’s experiments on *C. elegans* shed light on the importance of PCD, and they were awarded the 2002 Nobel Prize in Physiology or Medicine for their discoveries regarding “genetic regulation of organ development and programmed cell death” [97].

In 2011, Pourkarimi et al. showed that CED-9 and CED-4 mostly have different localizations in the *C. elegans* germline and stated that although there are some colocalizations between CED-9 and CED-4, it cannot describe the previously accepted model for apoptosis induction. CED-4 is mainly localized in the perinuclear space, whereas CED-9 is localized on the outer mitochondrial membrane. They proposed that apoptosis induction in *C. elegans* is more similar to that of the mammalian system than previously thought [90].

## 2. Apoptosis Induction and Regulation

Regardless of the proapoptotic stimuli, apoptosis is induced via two separate yet intertwined pathways, namely, intrinsic and extrinsic apoptotic pathways. In this section, the molecular mechanism and regulation of apoptotic pathways, from induction to execution, are discussed.

### 2.1. Intrinsic Pathway

The intrinsic pathway of apoptosis, also called the mitochondrial pathway, is initiated in response to physiological or pathological conditions. The mitochondria are the main source of energy in the cell. The intact inner membrane of the mitochondria is crucial to maintain the mitochondrial membrane potential (Δψ), needed for ATP synthesis. The mitochondrial permeability transition pore (mPTP) is a non-specific pore in the inner mitochondrial membrane; its opening leads to the swelling of mitochondria, rupture of the mitochondrial outer membrane, and the release of the proapoptotic factors such as cytochrome c [98,99,100,101]. The life-supporting cytochrome c, which is an electron transport chain member and crucial for generating ATP, activates the death pathway if released from the mitochondrial intermembrane space to the cytoplasm.

**Figure 4:**
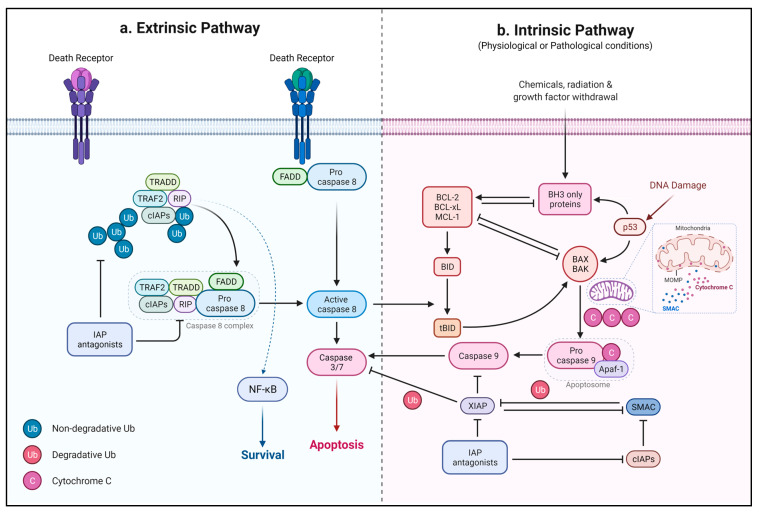
Extrinsic and intrinsic pathways of apoptosis. (**a**) Extrinsic pathway: following an external death signal, death receptors are activated by oligomerization, leading to a cascade of caspase activation and apoptosis. (**b**) Intrinsic pathway: several internal stimuli can activate the intrinsic apoptotic pathway, including chemicals, radiation, growth factor withdrawal, and genotoxic insults. The illustration is created with BioRender.com. BCL-2: B-cell lymphoma 2; BCL-xL: B-cell lymphoma-extra large; MCL-1: myeloid cell leukemia 1; BAX: Bcl-2-associated X protein; BAK: Bcl-2 antagonist/killer; BID: BH3 interacting-domain death agonist; tBID: truncated BID; XIAP: X-linked inhibitor of apoptosis protein; SMAC: second mitochondrial-derived activator of caspases; cIAPs: Cellular inhibitor of apoptosis proteins; FADD: Fas-associated death domain protein; TRADD: TNF-receptor-associated death domain; TRAF2: TNF-receptor-associated factor 2; RIP: receptor-interacting protein; NF-κB: nuclear factor kappaB.

The intrinsic or mitochondrial pathway initiates with the release of mitochondrial cytochrome c to the cytoplasm when triggered by proapoptotic stimuli such as DNA damage, accumulation of reactive oxygen species (ROS), endoplasmic reticulum (ER) stress, and DNA replication stress [102,103,104,105,106,107]. The release of cytochrome c from mitochondria is induced by proapoptotic members of the Bcl-2 family, such as BAX, BAK, and PUMA. BH3-only domain proteins are divided into activators of mitochondrial apoptosis, e.g., BIM, BID, PUMA, and NOXA, and sensitizers that act upstream of activators, e.g., BAD, harakiri, and BIK [108,109,110,111]. Direct binding of activators to BAX and BAK leads to their conformational changes and activation via oligomerization. Active BAK and BAX induce the formation and opening of mPTP, leading to mitochondrial permeability transition (MPT), mitochondrial outer membrane permeabilization (MOMP), and the release of cytochrome c [112,113,114,115]. MOMP is mainly considered the point of no return in cell death, since irrespective of the steps following MOMP, cells are mostly committed to death, even in the absence of caspases activity (Figure 4) [116,117,118].

Inhibitors of apoptosis (IAPs) are a group of proteins identified in different cancers that prevent apoptosis by blocking caspase activity [119,120,121,122,123,124]. Following MOMP, the second mitochondria-derived activators of caspases (SMACs) are released to the cytoplasm in addition to cytochrome c [125,126]. SMACs or direct IAP-binding protein with low pI, DIABLO, help induce apoptosis by binding to IAPs, blocking their apoptotic inhibitory effects [127,128,129,130,131].

Cohen’s lab and others showed that the released cytochrome c interacts with Apaf-1, ATP, and procaspase 9 to assemble the apoptosome, a caspase activating protein complex that converts procaspase 9 to active caspase 9 [87,132,133,134,135,136,137,138]. This conversion is mediated by either its autocleavage with caspase 9 at Asp-315, resulting in a 35 kD fragment (p12/p35), or caspase-3-mediated cleavage at Asp-330, which forms a 37 kD fragment (p10/p37) [139]. Caspase 9 initiates a cascade of caspase activation by converting procaspase 3 and 7 to their active forms [87,137,140]. Activated caspase 3 and 7 convert other procaspases to activated caspases, leading to the amplification of the apoptosis cascade [140,141]. For instance, a study of caspase 3 depletion revealed that caspase 3 starts a positive feedback loop by removing the inhibitor docking domain of caspase-9 and converting it to a p10 fragment; this fragment is not detected in caspase-3-depleted cells [140]. Further studies reported that XIAP, the X-linked inhibitor-of-apoptosis protein, interacts with a p12/p35 fragment of caspase 9, leading to its inhibition, whereas the p10/p37 fragment is merely inhibitable by XIAP [142,143].

### 2.2. Extrinsic Pathway

The extrinsic apoptotic pathway is initiated when death receptors (DRs) are activated via binding to soluble or cell surface ligands on other cells. DRs are transmembrane proteins of the tumor necrosis factor receptor superfamily (TNFRSF), which have a cysteine-rich extracellular domain and a cytoplasmic death domain. Interaction of receptors with their ligand leads to the oligomerization of the receptor’s cytoplasmic death domains, which activates the cell death cascade. Generally, the extrinsic pathway is induced by a group of ligands, including tumor necrosis factor (TNF), first apoptosis signal (or factor associated suicide) ligand (FasL), or TNF-related apoptosis-inducing ligand (TRAIL) (Figure 4) [144].

#### 2.2.1. Tumor Necrosis Factor Receptor (TNFR)

Cytokine TNF-alpha (TNF-α) is the main ligand for activating extrinsic apoptosis, predominantly made by active macrophages [145,146,147,148]. There are two types of TNF-α, namely, transmembrane TNF-α (tmTNF-α) and soluble form TNF-α (sTNF-α), that can be released via the proteolytic cleavage of transmembrane TNF-α [149,150]. TNF-α binds to two distinct receptors: TNF receptor 1 (TNFR1, also known as CD120a) and TNF receptor 2 (TNFR 2, also known as CD 120b) [151,152]. Although TNFRs 1 and 2 are functionally related, they belong to two distinct subfamilies of TNFRSF. Unlike TNFR 2, TNFR 1 contains a cytoplasmic death domain (DD) that serves as a platform for requiting other cytoplasmic DD-containing proteins. The molecular mechanism of action of the TNFR 1 signaling pathway is complex, with opposite physiological outcomes. TNFR 1 signaling predominantly serves as a prosurvival and proinflammatory signaling pathway, and, in opposition to TNFR 2, which is only expressed in immune cells, TNFR 1 is expressed in the vast majority of cell types. The prosurvival and proliferation signaling of TNFR 1 depends on the nuclear translocation of nuclear factor-kappa B (NFκB), a transcriptional factor that controls the expression of groups of cytokines and genes related to proliferation.

The interaction of TNF-α with TNFR 1 leads to conformational changes in TNFR 1 and, subsequently, the resale of SODD (silencer of death domains) from its death domain. In the absence of TNF-α stimulation, SODD interaction with TNFR 1 hinders the interaction of other cytoplasmic death-domain-containing proteins from preventing self-aggregation and spontaneous activation of the TNFR 1 proteins. Upon the release of SODD protein, the TNF-receptor-associated death domain (TRADD) binds to the DD. The binding of TRADD to TNFR 1 will initiate the recruitment of other molecules that are crucial for TNFR 1 activation, such as TNF-receptor-associated factor 2 (TRAF 2), receptor-interacting serine/threonine protein (RIP), a cellular inhibitor of apoptosis protein 1 and 2, cIAP1 and cIAP2, and RIP-associated ICH-1/CED-3-homologous protein with a death domain.

The function of TNFR 1 is through two consecutive signaling complexes [153]. Complex I is bound to the plasma membrane and consists of TNFR1, TRADD, receptor-interacting serine/threonine protein kinase 1 (RIPK1), TRAF2, cIAP1, and cIAP2. The assembly of complex I leads to the prosurvival pathway via the transcription factor NFκB. Post-translational modifications (PTMs) of TRADD and RIPK1 mediate their detachment from the receptor. Then, TRADD, RIPK1, Fas-associated death domain protein (FADD), and caspase-8 assemble to form cytoplasmic complex II, known as death-inducing signaling complex (DISC) [153,154,155,156,157,158]. In contrast, in the presence of activated NFκB, FADD-like interleukin-1beta-converting enzyme (FLICE)-inhibitory protein long-form (FLIP(L)) inhibits caspase-8, leading to cell survival. However, if complex I fail to activate NFκB, complex II activation leads to cell death [153,159,160]. Autocatalytic activation of procaspase-8 and procaspase-10 in complex II leads to the activation of effector caspases (3 and 7) in two ways: directly via the cleavage and conversion of procaspase to caspase or indirectly via crosstalk with the intrinsic pathway through activating BID [161,162,163,164,165,166,167,168]. It has been shown that FLIP inhibits death-receptor-induced apoptosis via its interaction with members of DISC, FADD, and FLICE [169,170,171,172,173]. Additionally, RIPK1 regulates the extrinsic apoptosis pathway by interacting with the death domains of FADD and TRADD in DISC [153,158,174,175]. The effects of RIPK1 are further regulated via PTM through the NFκB-mediated pathway or independently of NFκB [176,177]. Recently, Smyth et al. introduced FLIP(L) as a pseudocaspase, which has structural homology with caspases 8 and 10 in its C terminal; however, this homology domain in FLIP(L) lacks catalytic function. The canonical function of FLIP(L) in apoptosis is attributed to its N-terminal death effector domain (DED), which enables its recruitment to DISC. Additionally, FLIP(L) has been shown to have non-canonical cellular functions, mediated independently of caspase-8, such as its role in inflammation, autophagy, cell motility, and proliferation, all of which have important roles in cancer development and progression [178].

#### 2.2.2. TNF-Related Apoptosis-Inducing Ligand (TRAIL)

Death receptor 4 (DR4, TRAIL-R1) and DR5 (TRAIL-R2) are other members of TNFRSF that induce apoptosis when activated by TRAIL/Apo-2L, a member of the TNF family of cytokines [179,180,181,182,183,184]. Following the binding of TRAIL, FADD and caspase-8 are recruited to the receptor, and this assembly leads to caspase activation, MOMP, BID cleavage, and cytochrome c rearrangement [161,184,185].

#### 2.2.3. Fas Receptor

The cluster of differentiation 95 (CD95)/Apo-1/Fas is another member of TNFRSF that induces apoptosis when triggered by a homotrimer of the Fas ligand (FasL) [186,187,188,189,190,191]. Upon activation of Fas by FasL, the cytoplasmic death domain of Fas induces the assembly of FADD, caspase-8, and caspase-10 in the DISC complex [192,193,194,195,196,197]. DISC functions in two different types of cells: in type I, following the conversion of pro-caspase-8 to caspase-8, it directly interacts with other caspases and induces apoptosis; whereas in type II cells, DISC triggers an amplified cascade of caspase-8 activation via crosstalk with the intrinsic pathway and escalates the release of proapoptotic factors from mitochondria [186,198].

Caspases are highly conserved proteins that are key players in apoptosis signaling pathways and other biological functions, such as inflammation [199]. They are generally divided into three groups based on their similarity in sequence and function: Group I are inflammatory caspases consisting of caspases 1, 4, and 5; Group II are effector or executioner caspases comprising caspases 3 and 7; the third group is initiator caspases, which include caspases 2, 8, 9, and 10 [200,201]. Caspase 6 was classified as an executioner caspase for a long time based on its sequence; however, functional studies have proposed it to be an initiator caspase since its transient activation is insufficient for apoptosis induction [202]. Effector caspases are responsible for some of the morphological and biochemical features of apoptosis, comprising apoptotic body formation, DNA fragmentation, and exposure of phosphatidylserine (PS) [203,204,205,206,207,208,209,210,211]. Caspases usually are inactive and are activated via proteolytic cleavage. As discussed earlier, initiator caspases are activated by interaction with Apaf-1, which consequently activates effector caspases [212]. Additionally, the release of apoptosis-inducing factor (AIF) has been shown to induce caspase-independent apoptosis [213,214] (Figure 4).

## 3. Apoptosis in Human Diseases

Apoptosis is one of the most studied mechanisms in physiological and pathological conditions, and its precise regulation is crucial to human health. Failure to regulate apoptosis can lead to several diseases; increased apoptosis can lead to neurodegenerative and autoimmune diseases, whereas its downregulation could lead to cancer by assisting tumor cells in escaping cell death and developing drug resistance. Since apoptosis is ablated in most cancers, novel therapies target cell death mechanisms via either intrinsic or extrinsic apoptotic pathways [215]. Following the introduction of Bcl-2 in hematological malignancy by Vaux et al. in 1988, extensive research has been done to assess the role of Bcl-2 protein family members in mitochondrial apoptosis. It should be noted that the delicate balance between anti- and proapoptotic proteins of the Bcl-2 family determines the life and death decisions of cells. This balance is influenced by several factors, including interaction, localization, expression level, half-life, and PTM of Bcl-2 proteins [83,216,217,218,219,220]. During different stages of tumorigenesis and metastasis, cancer cells evade apoptosis by modulating Bcl-2 protein family members, such as by the upregulation of antiapoptotic Bcl-2 proteins and the downregulation or removal of proapoptotic Bcl-2 members [221]. Several events have been reported to be responsible for the upregulation of pro-survival Bcl-2 proteins; among the events is Bcl-2 translocation (first reported in follicular lymphoma), which is not prevalent among other cancers. As discussed earlier, Vaux was the first to report that the overexpression of antiapoptotic Bcl-2 is not enough for oncogenesis and to show its pro-survival function. Accordingly, the detection of translocation t(14;18) of Bcl-2 in healthy individuals, together with in vivo studies in mice, indicated that mimicking this translocation was minimally oncogenic, and several other findings have proven that Bcl-2 is not an oncogene [222,223]. Further studies reported that gene amplification, augmented expression, translation, and protein stability were responsible for higher antiapoptotic Bcl-2 in cancers [221]. Moreover, sensitizer proapoptotic Bcl-2 proteins, such as Bad, partake in apoptosis by inhibiting antiapoptotic proteins or controlling the cellular localization of BAX and BAK [81,110]. A recent paper by Soond et al. proposed a novel apoptosis regulation mechanism in which cathepsin S cleaves BAX, which could be of interest for cancer treatment [224]. On the other hand, p53 responds to stress conditions, such as DNA damage, by transcriptional activation of proteins that are essential for DNA repair, cell cycle arrest, and apoptosis [225,226,227]. p53 can also interact with the promoter region of proapoptotic Bcl-2 family members such as BAX to regulate their expression, leading to apoptosis regulation [228]. Accordingly, tumors harbor mechanisms to block apoptosis via inhibiting the tumor suppressor gene p53, and therapeutic modalities tend to inhibit these inhibitors [229].

Among the members of the extrinsic apoptosis pathway, TNFRs are the most attractive targets of cancer therapy [230]. Nowadays, death receptor agonists alone or in combination with other therapies are used in the clinical setting to treat cancer [231]. For instance, Masum et al. synthesized a luminescent iridium complex–peptide hybrid (IPH) to detect tumor cells and induce apoptosis by its peptide, which imitates TRAIL and integrates with death receptors [232,233]. Immune checkpoint inhibition and cell-mediated immunotherapy could also induce apoptosis through the extrinsic pathway [234].

A high rate of apoptosis leads to several diseases, including autoimmune, neurodegenerative, and inflammatory disorders. Unlike cancer therapies that tend to induce apoptosis, treatments for these pathologies are mostly aimed at apoptosis inhibition [235]. Additionally, some infectious diseases induce apoptosis in several human tissues, and treatments are aimed at apoptosis inhibition. For instance, severe acute respiratory syndrome coronavirus 2 (SARS-CoV-2) has been shown to induce apoptosis, necroptosis, and inflammation by activating caspase-8 in the lung epithelial cells, leading to lung damage and multi-organ failure in critically sick patients [236]. Additionally, the induction of these programmed cell death mechanisms has been mainly attributed to the death domain (DD) protein superfamily, and their inhibition has been proposed as a therapeutic target [237]. A very recent study showed that the highly pathogenic SARS-CoV-2 and middle east respiratory syndrome coronavirus (MERS-CoV) infections trigger the intrinsic apoptosis pathway by protein kinase R-like endoplasmic reticulum kinase (PERK) signaling. PERK regulates apoptosis through the proapoptotic Bcl-2 members BIM, PUMA, and NOXA. Although most viruses develop an apoptosis evasion mechanism to propagate within the host, surprisingly, it has been shown that apoptosis facilitates viral replication in MERS-CoV infection via caspase-mediated viral genome cleavage that helps virus production or activates host pathways, assisting viral propagation. Therefore, apoptosis inhibition could be a potential therapeutic mechanism in coronavirus disease (COVID-19) and MERS treatment [238]. However, cell death comes from different modalities, and apoptosis is no longer considered the only mechanism of programmed cell death. In 1973, Schweichel and Merker used electron microscopy to propose three types of cell death based on cell morphology, viz, Type I (apoptosis), Type II (autophagy), and Type III (necrosis) [239,240]. Although these three types are the best-known cell death mechanisms, they are not the only ones. Nowadays, more than a dozen types of regulated cell death (RCD) have been defined by the Nomenclature Committee on Cell Death (NCCD); the identification of their mechanisms could help to treat several disorders, including cancer and inflammatory, cardiovascular, and neurodegenerative diseases [241]. The last step in the apoptotic process is the engulfment of apoptotic cells by neighboring cells or macrophages. This is one of the most important connections between apoptosis and autophagy. In the following section, we deep dive into the history, morphological and molecular characteristics, and mechanism of autophagy as a concluding step in apoptosis and as a distinct type of cell death mechanism.

## 4. Autophagy

In the twentieth century, seminal studies by Christian de Duve led to the discovery of many previously unknown cellular organelles such as lysosomes. During his voyage into the field of the lysosome, he observed lysosome-like membrane-bound particles containing mitochondria and other cytoplasmic materials. The sequestered cytoplasmic materials, which contained acid phosphatase, similar to lysosomal vacuoles yet distinct from the typical lysosome, convinced De Duve to coin the term autophagy (ŏt-ō-fā’-jē, Greek: self-eating). It is worth mentioning that the lysosomal involvement in autophagy was yet to be discovered at the time. It was around the same time that Albert Claude mastered the preparation of biological samples for use in electron microscopy at the Rockefeller University (then the Rockefeller Institute), which enabled scientists to visualize subcellular structures with a resolution beyond that of the existing light microscope [242]. Using electron microscopy and cytochemistry studies, membrane-confined structures containing different organelles provided a basis for autophagy research and confirmed previous observations [243,244,245,246,247,248,249]. These early findings became the cornerstone of autophagy research and set the stage for understanding the molecular basis of autophagy. In 1974, De Duve, Albert Claude, and George Palade were awarded the Noble Prize in Physiology or Medicine for their discoveries concerning the structural and functional organization of the cell [250].

Now, we know autophagy as a tightly regulated cellular process in which cellular organelles and cytoplasmic materials are sequestered, with a double-membrane vesicle called the autophagosome, and digested via a lysosomal-mediated process. [251,252]. Autophagy is induced both in physiological and pathological conditions, and, over the years, electron microscopy and cytological investigation have shown that this process is affected by different factors. In one of the early studies, the perfusion of rat liver with glucagon increased lysosomal formation, and, in all instances, the lysosomal vacuoles contained mitochondria or at least remnants of them [244]. These observations led to identifying a stepwise process of mitochondrial engulfment and digestion within the lysosome [244]. Interestingly, it was established, in 1977, that insulin has an anti-autophagic effect, which validated the counteracting effects of insulin and glucagon [253]. In line with this finding, it was shown that autophagy is induced in the absence of serum and amino acids [254]. This increase in autophagy activity can be inhibited by insulin, amino acid supplements, or elevated levels of cAMP [255,256,257,258,259]. These were the initial key observations linking autophagy to diabetes. Autophagy has been shown to have a substantial role in the survival and function of insulin-producing pancreatic beta cells [260,261,262]. More specifically, lipophagy, a selective macroautophagy process, and general autophagy prevent Type II diabetes by regulating lipid metabolism and lipid droplet clearance [263,264].

Autophagy is not always induced following starvation; there is nutrient-independent autophagy, known as “quality control (QC) autophagy” or “basal autophagy”, which is responsible for removing damaged organelles and toxic protein aggregates from the cell, thereby maintaining cell homeostasis [265]. One of the key regulators of autophagy is the mechanistic Target of Rapamycin (mTOR) which was discovered in 1992. Rapamycin, a product of *Streptomyces hygroscopicus* bacteria, was first isolated by Sehgal [266]. It was initially developed for its antifungal activity but has become more known for its antiproliferative effect in a wide variety of cells, in addition to its immunosuppressive effects [267]. Research on the effect of the rapamycin drug by David Sabatini led to the biochemical purification of its target protein, the target of rapamycin (TOR) [268]. Independently, TOR was also identified in a yeast genetic screen in an attempt to isolate yeast mutants that are resistant to rapamycin [269]. Around the same time, other groups identified the TOR protein, and, by then, the name mTOR had been coined by Robert Abraham. mTOR was shown to be universally conserved in various organisms, from yeast to mammals [270,271,272,273,274,275]. The mammalian target of rapamycin, which recently has been referred to as the mechanistic target of rapamycin (mTOR), is a serine/threonine kinase with various effects on biological functions, mostly following stress [276]. mTOR is the catalytic subunit of two complexes with different sensitivity to rapamycin, namely, mTOR complex 1 (mTORC1) and mTOR complex 2 (mTORC2) [276]. Meijer’s group advanced the field by discovering the induction of autophagy by rapamycin [277]. They further showed that the PI3K protein family is central in autophagy, and PI3K inhibitors, such as wortmannin, LY294002, and 3-methyladenine, inhibit autophagy [278].

mTOR is a master regulator of nutrition signaling and is involved in various cellular pathways, including regulating protein synthesis, fine-tuning the metabolism, promoting cell growth, cell cycle progression, and autophagy in different model systems [276,279,280,281,282]. Ohsumi’s laboratory, in 1998, confirmed the described effects of rapamycin in the mammalian system by showing that mTOR also inhibits autophagy in yeast [259]. Deficiency in the gene coding for the *C. elegans* homolog of mTOR, *let-363*, has been shown to inhibit global mRNA translation, leading to developmental arrest and intestinal atrophy [283]. Interestingly, mTOR inhibition in *C. elegans* increases the worm’s lifespan through insulin signaling, mimicking the effect of starvation in the worm’s longevity [284,285]. Starvation-induced autophagy in *C. elegans* was studied following the discovery of genes involved in the insulin-like/IGF-1 signaling (IIS) pathway, including the insulin/IGF-1 receptor, *daf-2* (abnormal DAuer Formation), and *daf-16*, the Forkhead box protein O transcription factor (FOXO) [286,287,288].

## 5. Molecular Basis of Autophagy

Until 1992, the field of autophagy was prominently based on cytological studies; however, identifying autophagy vacuoles in *S. cerevisiae* became instrumental in uncovering its genetic regulation. Yeast contains a vacuole, the only yeast organelle readily visible using a phase-contrast microscope, which poses a similar characteristic as the mammalian lysosome. Upon various starvation conditions such as nitrogen starvation, multiple spherical structures form and fill the vacuole. Electron microscopic investigation of the vacuoles indicated the presence of cytoplasmic materials such as the endoplasmic reticulum, mitochondria, and ribosomes, the hallmark of autophagy activity [289]. Yoshinori Ohsumi’s group profoundly contributed to our current knowledge on the genetic regulation of autophagy and performed the first genetic screen to find autophagy-defective mutants in yeast [290]. Accordingly, similar screens identified other autophagy-related genes, with each research group naming the identified genes differently. In 2003, Klionsky et al. reviewed all identified genes related to autophagy and proposed a standard nomenclature, in which the gene name is *ATG* and the protein name Atg, meaning AuTophaGy-related [291,292]. The massive amount of research on the molecular aspects of autophagy is indebted to the discovery of *ATG* genes in the yeast system. In 2016, Yoshinori Ohsumi won the Nobel Prize in Physiology or Medicine for discovering autophagy mechanisms [293].

Using yeast two-hybrid screening, Levine’s group identified *beclin1* as a novel Bcl-2-interacting protein [82]. Soon after, it was shown that *beclin1* is a tumor suppressor gene and the mammalian homolog of yeast *ATG6*. The allelic deletions of *beclin1* are reported in human breast, ovarian, and prostate cancer and are believed to induce autophagy and inhibit tumorigenesis [294,295]. Further in vivo studies confirmed *beclin1* as a haploinsufficient tumor suppressor gene contributing to embryonic development and autophagy. These studies confirmed autophagy as a mechanism that regulates cell growth and proliferation and, if defected, contributes to human cancers [296,297]. A 2005 paper by Levine’s group showed that the antiapoptotic protein Bcl-2 inhibits autophagy by interacting with *beclin1* and could protect cell death by regulating autophagy levels corresponding to cell survival [298]. Given that autophagy-related genes are evolutionarily conserved, *C. elegans* counterparts of yeast and mammalian ATGs were identified via genomic homology [299,300]. One of the earliest studies on this was the work by Melendez et al., who identified *bec-1*, the worm’s counterpart of mammalian *beclin1*/ATG6, which is important for *C. elegans* life extension [301]. Interestingly, in line with the pro-survival function of *beclin1* in mammalian cells, the inactivation of *C. elegans bec-1* induces caspase-dependent cell death. Additionally, the antiapoptotic CED-9/Bcl-2 physically interacts with BEC-1 in nematodes [302].

## 6. Different Types of Autophagy

**Figure 5:**
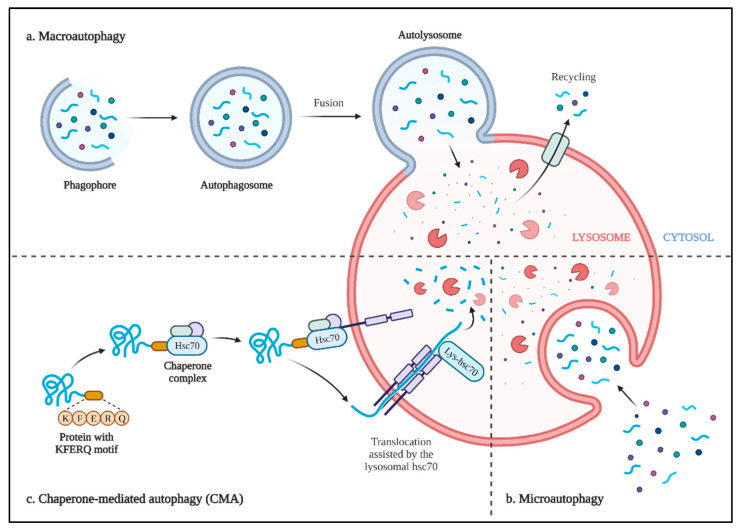
Three different types of autophagy. This schematic figure describes different types of autophagy. (**a**) Macroautophagy: a phagophore initiates by the assembly of several proteins, and the membrane sequesters the degradation target during autophagosome maturation. Following autophagosome and lysosome fusion, autolysosome forms and degradation starts with lysosomal proteases. (**b**) Microautophagy: some cytoplasmic materials are directly uptaken by lysosomes via the assistance of SNARE and ESCRT protein complexes. (**c**) Chaperone-mediated autophagy (CMA): this specific type of autophagy is specialized to degrade proteins with a KFERQ motif. This process is mediated by the chaperone complex and several proteins and is the only type of autophagy that is independent of vesicles. Targeted proteins are translocated to the lysosome via lysosomal membrane proteins. Regardless of the autophagy pathway, degraded materials are transferred to the cytosol by lysosomal membrane proteins, mainly permeases, to be recycled in other procedures. The illustration is created with BioRender.com. SNARE: soluble N-ethylmaleimide-sensitive factor attachment proteins receptor; ESCRT: endosomal sorting complex required for transport; Hsc70: heat shock cognate 71 kDa protein; K: lysine; F: phenylalanine; E: glutamic acid; R: arginine; Q: glutamine.

Generally, there are three broad types of autophagy in the mammalian system: macroautophagy, microautophagy, and chaperone-mediated autophagy (CMA) (Figure 5).

### 6.1. Macroautophagy

**Figure 6:**
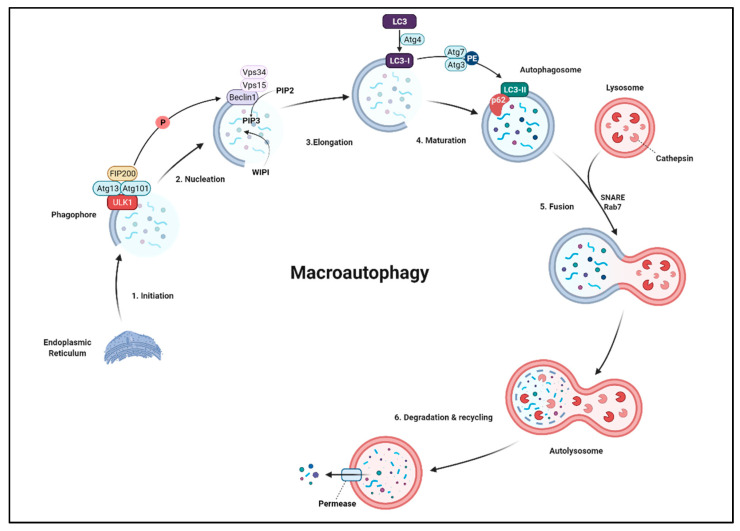
Macroautophagy: There are several steps in the formation of a phagophore, its maturation into an autophagosome, and, finally, fusion with the lysosome to degrade the cargo and recycle macromolecules for further use. The illustration is created with BioRender.com.

Macroautophagy, which is mainly used to describe autophagy, is a stepwise procedure of self-cannibalism that consists of the induction, nucleation, elongation, maturation, and closure of the phagophore, leading to autophagosome formation, fusion with the lysosome, and, finally, degradation of sequestered organelles; several proteins take part in the process and regulation of each step (Figure 5a and Figure 6) [303,304]. Given that autophagosome formation is the most important characteristic of macroautophagy, the process is executed and regulated by the core molecular machinery of autophagy, which consists of six indispensable functional groups, viz, the autophagy initiation complex (AIC), vesicles containing Atg9, the phosphatidylinositol 3-kinase (PI3K) complex, the Atg2-Atg18 complex, and two conjugation systems, namely, Atg8 and Atg12 [305].

#### 6.1.1. Initiation

Autophagy is induced by various extrinsic and intrinsic pathways and transmitted through different signaling cascades such as mTOR1, the main nutrient-sensing signaling molecule, which is a converging hub for several signaling pathways [306,307]. TORC1 inactivates autophagy by inhibiting the formation of the Atg1 complex, mediated by direct hyperphosphorylation of Atg13 by TORC1. In contrast, conditions such as starvation inhibit TORC1 activity, leading to autophagy induction by initiating AIC or Atg1 complex assembly. In yeast, the Atg1 complex consists of Atg1, Atg13, Atg17, Atg29, and Atg31 [259,308,309]. Atg13 is the regulatory subunit in this complex, and its interaction with Atg1 is necessary for Atg1 complex formation [310]. In the absence of TORC1 activity, Atg13 is rapidly dephosphorylated and triggers Atg1 complex formation. Atg1 is a serine/threonine protein kinase, initially cloned in yeast by Ohsumi’s group as a homolog of *C. elegans* UNC-51 [308]. Later, ULK1 (UNC-51-Like Kinase 1) and ULK2 were cloned in mice and humans and identified as the ULK protein kinase family. These proteins have proline/serine-rich (PS) domains that have shown to be evolutionarily conserved in eukaryotes [311,312,313]. Assembly of several Atg1/ULK1 complexes provides a scaffold to recruit other core Atg proteins required for autophagy initiation [314,315,316,317].

mTOR-mediated autophagy in the mammalian system is mainly induced and regulated through the ULK1-ATG13-FIP200 complex [318,319]. FIP200, a focal adhesion kinase family interacting protein of 200 kD, is an ULK1-interacting protein whose interaction is important for ULK1 stability and phosphorylation [320]. Atg101 is another mammalian protein interacting with the ULK1-ATG13-FIP200 complex through Atg13, and this interaction is important for the stability and phosphorylation of both ULK1 and FIP200 [321].

In 2011, Kim et al. reported a regulatory mechanism of autophagy via the ULK1 phosphorylation of different serines. They showed that AMP-activated protein kinase (AMPK) phosphorylation of ULK1 at Ser 317 and Ser 777 induces autophagy, while mTOR-induced phosphorylation at Ser 757 blocks ULK1-AMPK interaction, preventing autophagy, and proposed a synchronization between these kinases [322]. Additionally, there are several activation/inhibition loops via phosphorylation. For instance, AMPK inhibits mTORC1 directly and indirectly and inhibits starvation-induced autophagy via phosphorylation of Atg13 [323]. mTORC1-induced phosphorylation of Atg13 inhibits ULK1/2, while ULK1/2 phosphorylates mTORC1 and AMPK [324,325]. Recently, a computational model has been proposed to regulate cell metabolism via an interaction between internal and external signaling pathways involving mTORC, AKT, and AMPK [326]. There are also TOR-independent regulations of autophagy. The noncanonical regulatory mechanisms of autophagy have been reviewed by Corona Velazquez and Jackson [307].

#### 6.1.2. Nucleation

The next step of autophagy is assembling membrane portions to form a phagophore, a cup-shaped structure. Contrasting evidence suggests that the origin of phagophore double-layered membranes is either de novo assembly or organelles and the plasma membrane [327,328]. Generally, the phagophore nucleates from small vesicles containing transmembrane Atg9, which is required for interaction with the Atg1 complex [329]. Most of the core autophagic machinery proteins assemble at specific sites: either the phagophore assembly site (PAS) in yeast or autophagosome formation sites in other organisms [330]. Omegasomes (Ω), which are portions of the endoplasmic reticulum, enriched in phosphatidylinositol 3-phosphate (PI3P), are the mammalian counterparts of PAS [331]. Atg9 trafficking is regulated at different levels by several proteins, such as Rab GTPases [332,333]. Atg9 phosphorylation via the Atg1 complex results in the recruitment of Atg8 and Atg18, which facilitate the Atg9–Atg18 interaction, leading to phagophore biogenesis [334].

ULK1-mediated activation of Beclin1 (BECN1) via phosphorylation detaches the BECN1–Bcl-2 complex and induces the activity of PI3K complex I, consequently leading to autophagy [335,336,337]. Beclin1 is a PI3K complex I subunit, a class III PI3K consisting of Vps34, Vps15, Atg6 (BECN1), Atg13, and Atg38. PI3K complex I participates in autophagosome formation by phosphatidylinositol-3-phosphate (PtdIns(3)P or PI3P) production in autophagosome membranes and PI3P-binding protein recruitment [338]. PI3K complex I is regulated via interaction with several proteins, such as Bcl-2 and BIF-1 (Bax-interacting factor 1) [304]. It is worth mentioning that two independent classes of PI3K have opposing effects on autophagy [278]. As mentioned above, class III PI3K produces PI3P, which is required for autophagy, while class I PI3K produces PtdIns(3,4,5)P_3_ (PIP3), which inhibits autophagy [339]. Additionally, overexpression of PTEN, phosphatase, and the tensin homolog deleted on chromosome 10 has been shown to induce autophagy via reducing the PIP3 level [340]. The process of PI3P production is considered the nucleation step of autophagy.

#### 6.1.3. Elongation

Recruited PI3P-binding proteins are WIPI (Atg18), p62, LC3-1, and the Atg12 ubiquitin-like protein (UBL) conjugation system. WIPI (WD-repeat protein interacting with phosphoinositides) is recruited following increased concentrations of PI3P in preautophagosomes and promotes the elongation of phagophores [316]. p62, also known as sequestosome 1 (SQSTM1), is a multipotent ubiquitin-binding protein and a selective autophagy receptor. It plays a crucial role in the selective autophagy of ubiquitinylated proteins, targeting them to the autophagosome through its interaction with LC3 protein and mediating cargo uptake [341]. Selective autophagy is regulated through the PTM of selective autophagy proteins such as LC3 and p62 [342].

The other recruited proteins are the E3-like Atg5–Atg12–Atg16 complex, members of the Atg12 UBL conjugation system, which helps to target specific proteins to the developing autophagosome [343]. UBLs are small proteins conjugated through their carboxyl termini to the amino groups of the target molecules. This conjugation is mediated by E1, E2, and E3 enzymes, an activating enzyme, a carrier enzyme, and a protein ligase, respectively [344,345].

Overall, there are two UBL conjugation systems in the autophagy process: Atg8 and Atg12 [346]. *ATG5* and *ATG12* were the first mammalian autophagy genes identified by Ohsumi’s group in 1998. These genes encode UBL proteins, which conjugate to make a distinct covalent modification system necessary for autophagy, a conserved mechanism from yeast to humans [347]. This conjugation is mediated via ATG7, E1-like, and ATG10, which are E2-like proteins [348,349].

The other UBL in the autophagy mechanism is Atg8, which has been shown to have a physical interaction with Atg3, a specific E2-enzyme [350,351,352,353]. Consequently, Atg8 modification by Atg4 facilitates its binding to membrane phosphatidylethanolamine, and this protein–lipid conjugate is essential in membrane dynamics throughout autophagy [346,354]. LC3 (microtubule-associated protein-1 light chain 3 or MAP1LC3) was previously proposed to regulate neuronal microtubules. In 2000, Yoshimori’s group reported LC3 as the mammalian functional homolog of Atg8 and showed that it is processed into LC3-I and, subsequently, to LC3-II, which are particularly associated with autophagosome membranes. They proposed LC3-II as the first molecular marker of autophagosomes [355,356]. Before 2000, morphological analysis was the only way to detect autophagosomes, and the introduction of LC3-based assays was a turning point in the history of autophagy research in higher eukaryotic systems [357]. Atg4-mediated cleavage of LC3 to LC3-I, which leads to the recruitment of LC3-I to the autophagosome, is essential in the progression of autophagy [358,359]. LC3-II, a lipidated form of LC3, is produced by the conjugation of LC3-I to phosphatidylethanolamine (PE) via Atg7 and Atg3 on the autophagosome membrane, which is an essential step leading to autophagosome maturation (see below) [354,356].

#### 6.1.4. Maturation

Compared to the other steps in the autophagy process, autophagosome maturation is more complex and less understood. As mentioned above, LC3-I to LC3-II conversion leads to membrane growth and expansion in the presence of PI3P. However, the level of PI3P should be highly regulated as studies have shown that its high concentration inhibits autophagosome maturation and its fusion with lysosomes. Accordingly, it has been suggested that excessive PI3P removal by phosphoinositide phosphatases and the unconjugation of LC3-PE by the Atg4 protein family mediate the release of Atg proteins from the autophagosome membrane, leading to its maturation [360,361].

#### 6.1.5. Fusion

Mature autophagosomes containing the degradation target will fuse with lysosomes via SNAREs (soluble N-ethylmaleimide-sensitive factor attachment protein receptors) and Rab7 [362,363,364]. Following membrane fusion, the autophagosome releases its content to the lysosome to form an autolysosome [365]. The autolysosomal contents are then degraded using various lysosomal-associated proteases, such as cathepsins B and L [366]. After cargo digestion in the lysosomes, which is the concluding step of the autophagy process, several permeases, the lysosomal transmembrane transport proteins, recycle the macromolecules to the cytoplasm [367]. There are also other types of autophagy that share ATG proteins with macroautophagy, including the cytoplasm-to-vacuole targeting (Cvt) pathway, mitophagy (selective degradation of mitochondria), and pexophagy (selective degradation of peroxisomes) [368,369,370,371,372]. In contrast to the catabolic role of autophagy, the Cvt pathway is utilized to deliver vacuole-related proteins such as hydrolyses, an interesting observation initially detected in *S. cerevisiae*. The Cvt pathway is unique in the sense that it acts as a biosynthetic route for vascular enzymes (Figure 5) [373,374,375,376].

### 6.2. Microautophagy

Microautophagy, the direct engulfment of cytoplasmic materials by lysosomes, is involved in various cellular pathways, including quality control, biosynthetic transport, organelle remodeling, and metabolic adaptation [377,378]. Two protein complexes mediate microautophagy, namely, SNARE proteins and ESCRT (endosomal sorting complex required for transport) proteins, facilitating fusion-type microautophagy and fission-type microautophagy, respectively [378,379,380] (Figure 5b).

### 6.3. Chaperone-Mediated Autophagy

CMA, the third type of mammalian autophagy, is a selective specialized protein degradation mechanism, which, unlike other types of autophagy, is independent of vesicle formation [381]. For a protein to be selectively degraded via the CMA pathway, it needs to have the pentapeptide motif of KFERQ in its protein sequence [382]. CMA has been shown to participate in both physiological and pathological situations, including the cell cycle, maintaining hematopoietic stem-cell function, aging, neurodegenerative diseases, and cancers [383,384,385,386,387,388,389]. CMA is also regulated at different levels, from cell membrane proteins to chaperone proteins in the cytoplasm, through Lamp2a, Hsc70, GFAP, EF1α, and the mTORC2/PHLPP1/Akt signaling axis [390,391,392]. Recently, Miller’s group reported increased CMA following the inhibition of class I PI3K [393] (Figure 5c).

## 7. Autophagy in Health and Disease

The available evidence suggests the role of autophagy in preserving the genetic integrity of mitochondrial DNA, especially following aging-related accumulation of mutations in mtDNA due to a weak DNA repair mechanism in the mitochondria compared to the nucleus [394,395,396,397]. In 2007, White’s laboratory showed that autophagy is a cell guarding mechanism during metabolic stress against chromosome instability and cancer development [398]. Apoptosis and autophagy contribute to cellular homeostasis in normal conditions. Li et al. showed that in cancer cells defective in apoptosis, Beclin1 is not cleaved by caspase and increased autophagy leads to cancer cell survival [399]. They proposed Beclin1 as a target for blocking autophagy and sensitizing cancer cells to chemotherapeutic drugs [399,400]. Recently, overexpression of p62, a selective autophagy receptor, has been shown to be associated with giant cell tumor of bone (GCTB) recurrence [401].

Organelle-specific autophagy is essential for organelle structure and functions as a quality control mechanism in maintaining homeostasis, and its malfunction is related to the pathogenesis of several diseases, including inflammatory, cardiovascular, neurodegenerative, and autoimmune disorders [402,403]. Rubinsztein’s group reviewed the role of PTM of proteins via acetyltransferases and deacetylases in the regulation of autophagy, finding some connection with neurodegenerative diseases [404]. Other groups evaluated the regulation of autophagy in cancer and other diseases [405,406].

Recently, it has been shown that glycogen synthase kinase 3 beta (GSK3β) regulates and induces autophagy flux via ULK1 phosphorylation, which might be responsible for tumorigenesis [407]. Additionally, ULK1 has been shown to regulate cell cycle and chromosome segregation, and its deletion increases the genomic instability that can significantly reduce tumor progression [408]. p62 has several cellular functions, including autophagy, apoptosis, inflammation, and metabolism, and it largely contributes to human diseases such as cancer and neurodegenerative disease [409]. Autophagy has been shown to protect against neurological diseases [410,411]. In vivo studies have confirmed the role of autophagy in continuous cytosolic protein clearance as a preventive mechanism against neurodegenerative diseases [412,413]. Rubinsztein’s group demonstrated that activation of autophagy following mTOR inhibition by rapamycin could reduce aggregate-prone proteins and increase the removal of the aggregates in protein conformational disorders (PCD), such as Huntington’s disease (HD), suggesting the clinical application of rapamycin in HD [414]. Other studies also suggested that the inhibition of autophagy could induce aging- and age-related neurodegenerative diseases [415,416,417,418,419,420].

Molecular studies of the human genome have shed light on the role of autophagy in several human diseases. Analyzing pathogenic single nucleotide polymorphisms (SNPs) has revealed the association of autophagy-related genes with human diseases such as cancer, neurodegeneration, inflammatory, and lysosomal storage disorders (LSDs) [421]. In a recent study, Du et al. identified autophagy-related genes whose differential expressions are linked to breast tumors and constructed autophagy-related gene signatures with great prognostic value [422]. In addition, autophagy-related long non-coding RNAs (lncRNAs) have been identified as signatures for cancer prognosis and treatment targets, especially in breast, lung, and colorectal cancer [423,424,425,426,427,428,429,430,431]. Moreover, autophagy-related non-coding RNAs have also contributed to diagnosing and treating cardiovascular diseases [432,433].

A plethora of studies has suggested the role of autophagy in innate immunity to protect cells against host infection by intracellular bacteria and viruses, a type of selective macroautophagy called xenophagy. Some pathogens have developed mechanisms to evade or control autophagy for their infection [82,434,435,436,437,438,439,440,441,442,443]. Moreover, it has been shown that autophagy plays a role in adaptive immunity by increasing the presentation of antigens on major histocompatibility complex (MHC) class II molecules upon pathogenic infection, highlighting the role of autophagy in the T-cell-mediated immune response [444,445,446,447,448]. Recently, several research groups studied the interaction between autophagy and severe acute respiratory syndrome coronavirus 2 (SARS-CoV-2) infection, providing contrasting evidence on the role of autophagy in preventing the infection and mechanisms by which the virus evades autophagy and suggesting potential treatment targets based on the regulation and modulation of autophagy against the viral infection and its spread [449,450,451,452,453,454,455,456,457,458,459,460,461,462].

LSDs are rare, inherited metabolic diseases caused by malfunctioning autophagic and lysosomal degradation pathways that lead to the aggregation of undigested proteins in lysosomes, resulting in cell dysfunction. Therefore, autophagy modulation is considered a promising therapeutic mechanism for the treatment of LSDs [463,464].

Since autophagy is a key mechanism in cell homeostasis and its malfunction is associated with various human diseases, it must be tightly regulated. Sirtuins are a group of evolutionarily conserved deacetylase protein families with regulatory functions in metabolic and aging processes [465]. They modulate post-translational modification via lysine deacetylase or mono-ADP-ribosyl transferase activities on proteins functioning in several processes in physiological as well as pathological conditions, including but not limited to stress response, apoptosis, and inflammation [466,467]. Additionally, different members of the sirtuins family have been shown to regulate autophagy, mainly protecting against metabolic and age-related diseases, including cancer, cardiovascular, and neurodegenerative disorders, thereby making them a potential therapeutic target [467,468,469,470].

In 2004, Sugawara et al., by reporting the crystal structure of LC3, the mammalian homolog of yeast Atg8, as the first autophagy-related protein structure, contributed significantly to autophagy research [471]. Structure determination helps function prediction through several experimental and computational methods, including protein–protein interaction, and cumulative data lead to the design of new drugs that can modulate autophagy to treat diseases [472,473,474].

## 8. Crosstalk between Apoptosis and Autophagy

Until 1972, when Kerr coined the term apoptosis, cellular death fell solely under necrosis. However, research in the following decades has broadened our understanding of different death modalities. Apoptosis is now referred to as caspase-dependent programmed cell death to differentiate it from other modalities of cell death, such as PCD conferred by autophagy. As mentioned above, autophagy is induced as part of normal cellular hemostasis or by external stimuli. However, autophagy’s role in cellular remodeling has been shown in different organisms, especially as a protective mechanism during starvation and other cellular stress conditions. Autophagy can act as a double-edged sword in anti- and pro-cell survival depending on the physiological and pathological conditions. Apoptosis and autophagy have a very complex relationship, with complicated crosstalk. Many genes linked to apoptosis regulation have turned out to be also involved in autophagy. In many instances, autophagy precedes apoptosis, as a non-lethal dose of stress can initially induce autophagy. However, a cell’s prolonged exposure to stress can lead to suicidal death. The tumor suppressor protein, P53, is known to be the guardian of the genome, and mutation in P53 provides a fundamental advantage to cancer cells. Normally, the P53 protein is partially localized at a low level in the cytoplasm, but upon genotoxic insult or stress conditions such as hypoxia, the phosphorylated form of P53 translocates to the nucleus where it initiates its antiproliferative function by activating variable biological outputs such as cell cycle arrest, senescence, and apoptosis. Nuclear translocation of P53 results in its cytoplasmic reduction, and recent studies indicate that cytoplasmic P53 suppresses autophagy. Therefore, depletion of P53 beyond a threshold activates autophagy in the cytoplasm. Chemical inhibition of P53 by pifithrin or knockout of wild-type P53 increases autophagy in various cell lines [475]. Additionally, the expression of P53 protein lacking a nuclear localization signal (NLS) in the knockout background suppresses ectopic autophagy [475]. Mutants of P53 that are predominantly cytoplasmic inhibit autophagy, while the constitutive nuclear P53 mutants fail to suppress it [476]. Recent data suggest that the cytoplasmic P53 inhibits the ULK1–ATG13–ATG101–FIP200 complex and blocks autophagosome formation, tempering autophagy activity [477,478]. In line with these data, CEP-1 (the functional orthologue of P53 in *C. elegans*) inactivation induces autophagy in worms [479].

As stated above, p53 and autophagy have an intertwined connection. Nuclear P53 was shown to transcriptionally activate autophagy-related genes such as the genes involved in lysosomal maturation [480]. One example of the autophagy-related target genes of P53 is DRAM1 (DNA damage-regulated autophagy modulator 1), which encodes for the lysosomal membrane protein responsible for activation of the lysosome. Furthermore, other studies have suggested that key autophagy regulators are the target of P53. ChIP-seq combined with RNA sequencing upon DNA damage revealed a number of P53 targets that act upstream in the autophagy pathway, such as ULK1 and ULK2, linking DNA damage response to autophagy induction. mTOR, the master regulator of autophagy, is the nutrition-sensing molecule whose activity leads to multiple physiological outputs. mTOR is the integral component of two distinct yet related complexes, TORC 1 and TORC 2. TORC 1 acts downstream of PI3K/AKT, a transducing pathway that governs cell growth, proliferation, and survival by indirectly activating mTORC 1 [481]. Over the past years, it was well established that P53 can inhibit AKT activity, which results in the indirect inactivation of mTOR, promoting autophagy [482].

**Figure 7:**
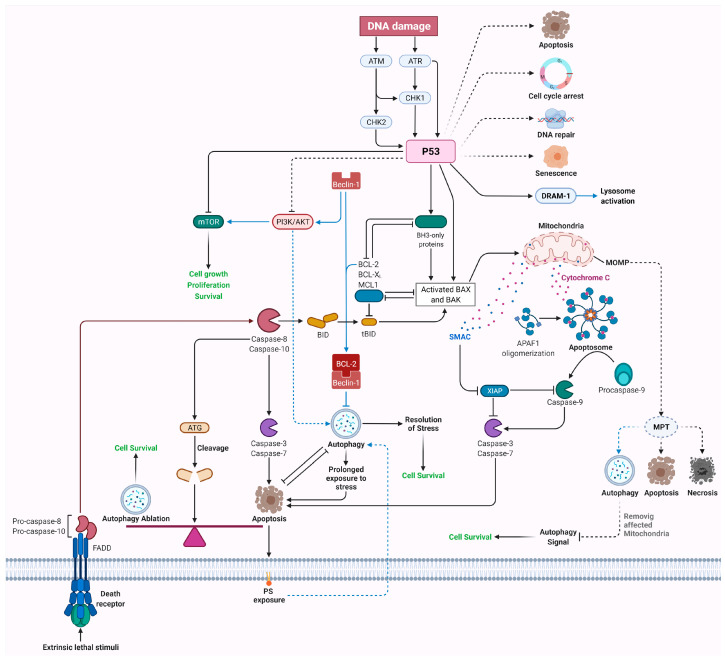
Crosstalk between apoptosis and autophagy. There is complex crosstalk between these two pathways, and they interact in different ways and situations. P53 is the master regulator of cell death and is activated via various cellular inputs, e.g., in response to DNA damage. Activation of P53 leads to apoptosis while inhibiting autophagy. Additionally, apoptosis can activate or inhibit autophagy based on the cell status. The mitochondrial outer membrane potential is disrupted upon activation of the intrinsic apoptosis pathway, leading to MPT. In this situation, the number of affected mitochondria determines the cell fate. If only a few mitochondria are affected, autophagy activity can eliminate them, leading to cell survival. However, when there are more mitochondria affected, apoptotic death will be the cellular fate. In certain situations, when the majority of mitochondria are affected by MPT, the cell undergoes necrosis. In addition, caspases are major proteins in apoptotic pathways whose enzymatic activity can activate the autophagy pathway by cleaving the precursor of ATG proteins or suppress autophagy by cleaving ATG proteins. Autophagy, in turn, could activate or suppress apoptosis. For instance, phosphatidylserine exposure following apoptosis could be a save-me or eat-me signal which activates autophagy. The stress level could tip the balance between life and death through autophagy activity. Autophagy can save cells by cleaning malfunctioning proteins, organelles, and so forth, or, upon prolonged exposure to stress, it can activate cell death. DRAM-1: DNA damage-regulated autophagy modulator 1; MOMP: mitochondrial outer membrane permeabilization; MPT: mitochondrial permeability transition; SMAC: second mitochondria-derived activators of caspases; FADD: Fas-associated death domain protein; PS: phosphatidylserine. The illustration is created with BioRender.com.

As mentioned previously, Beclin1 was initially discovered in a yeast two-hybrid screen as an interacting partner of the antiapoptotic Bcl2. Later, Beclin1 was also identified as an autophagy regulator, linking autophagy to programmed cell death. Consistent with the data, Beclin1 homozygous mutation in mice causes embryonic lethality, possibly due to the defect in autophagy regulation, while mice carrying homozygous mutations have an increased likelihood of spontaneous tumorigenesis [296,297]. Beclin1 acts as a platform for the binding of other autophagy regulators and binds to PI3KC3, VPS34 (a class III phosphoinositide kinase catalytic subunit), and the regulatory subunit VPS15/PIK3R4 to facilitate phagosome formation. The binding of Beclin to Bcl2 effectively diminishes autophagy. In addition, a Bcl2 mutant, defective in binding to beclin1, failed to inhibit autophagy (Figure 7).

As we have discussed, one of the best examples of the crosstalk between autophagy and apoptosis is the externalization of the membrane phosphatidylserine (PS) in apoptotic cells as a signal for their engulfment via autophagy; this process is called efferocytosis, and its malfunction leads to several disorders, including inflammatory and autoimmune diseases [483,484,485]. PS externalization is not particular to apoptosis, and it has been shown in other types of cell death, including necrosis. It has been suggested that based on the cell type, PS exposure could be an “eat-me” or “save-me” signal and is a physiological mechanism for cell clearance evolutionarily conserved from nematodes to humans [486].

We have also elaborated on the role of mitochondria and MPT in cellular functions, especially apoptosis. Lemasters et al. reviewed the effects of MPT in physiological and pathological conditions [395,487]. They mentioned that mitochondria have a half-life of 10–25 days in non-proliferating cells and provided evidence for the role of MPT in mitochondria turnover [488,489,490,491]. Using high-resolution microscopy, it has been shown that starvation-induced autophagy dramatically increases the number of depolarizing mitochondria [492]. Several factors may lead to MPT, including the accumulation of intracellular Ca^2+^, phosphate, and reactive oxygen species (ROS) and a decrease in pH [493]. Lemasters et al. also proposed that MPT is responsible for mitochondrial membrane depolarization and the signaling initiation of autophagy [395]. Nonetheless, autophagy is responsible for removing damaged or excess organelles in the cell, comparable to apoptosis at the tissue level; therefore, the level of MPT defines the type of death. When some mitochondria undergo MPT, autophagy is activated, and the affected mitochondria are removed, suppressing the autophagy signals. However, if the number of affected mitochondria is higher, autophagy may no longer be enough to answer the proapoptotic signals released from the mitochondria, and MPT leads to apoptosis induction. In the worst case, if almost all of the mitochondria are involved in MPT, oxidative phosphorylation uncoupling causes ATP depletion, which results in necrosis [395,487]. This is one of the most compelling hypotheses that beautifully relate the three well-known types of cell death to MPT. It has been suggested that autophagy and apoptosis act in synchrony such that, in normal conditions, autophagy functions as a cell guardian, but it can lead to cell death in case of extreme damage or apoptosis malfunction [494,495].

Although there are some caspase-independent pathways of apoptosis, caspases are the most important players in the apoptosis pathway. It has been shown that apoptosis crosstalks with autophagy through the recognition and cleavage of ATG proteins by caspases [496,497,498,499,500,501,502,503,504]. This cleavage could have two opposing results: ATG cleavage results in autophagy ablation as a protective mechanism against cell death or tips the homeostatic balance in favor of apoptosis [505,506,507,508,509]. This process is regarded as adherence of a dying cell to apoptosis by ceding other cellular functions [510]. However, it has been shown that ATG fragments can play different roles than their original functions by affecting autophagy and apoptosis [511,512]. Nevertheless, induction of autophagy via caspase has been described in some studies [512,513,514,515]. Autophagy, in turn, has been shown to have cell survival function by the degradation of proteins in the apoptotic pathway and the prevention of cells from entering suicidal states [516].

Levine and colleagues defined autosis in 2013 as a type of autophagy-dependent cell death that is independent of apoptosis and stimulated by starvation, neonatal cerebral hypoxia–ischemia, and autophagy-inducing peptides such as Tat-Beclin1 [517]. Autosis occurs due to severe autophagy and has its specific morphological characteristics, including an enhanced number of autophagosomes, nuclear expansion, inflation of the perinuclear space, and fragmentation of the endoplasmic reticulum [517,518,519,520].

## 9. Conclusions

Cell death is a highly regulated and essential process to maintain tissue integrity and homeostasis in an organism. Apoptosis, often referred to as programmed cell death, is a predefined and caspase-dependent cell death pathway. The two types of apoptotic pathways, intrinsic and extrinsic, work synergistically to ensure that the body removes only defective cells without increasing proinflammatory proteins. In contrast to apoptosis, necrosis is the uncontrolled cell death mechanism that implicates the upregulation of several proinflammatory proteins and contributes to the damage of surrounding cells. While autophagy involves the degradation of cellular components, being sequestered by the lysosomes, it either protects the cell from apoptosis or promotes it, depending on the stimuli. All forms of cell death are executed via different yet coinciding signaling pathways, and depending upon the cell status, they can be activated simultaneously. The disruption in the cell death pathways or the irregular activation of them can lead to various pathologies. In cancer, the uncontrolled cell division and growth due to the upregulation of survival genes and downregulation of antiapoptotic genes trigger tumor formation, whereas increased apoptosis can cause autoimmune diseases. Similarly, neurodegenerative conditions such as Parkinson’s result from the failure of autophagy, which results in the neuronal accumulation of protein aggregates. Hence, an intricate balance must be maintained between cell survival and cell death. Until now, there are many cell death genes whose functions have been explored, but, for some, the functions’ comprehension remains elusive. The tissue and organ-specific activation of cell death pathways affect how different illnesses respond to therapies. Therefore, a deep understanding of cell death mechanisms is vital in developing therapies and drugs against cancer and other diseases.

## 10. Materials and Methods

### 10.1. Developmental Apoptosis

The *C. elegans* N2 Bristol strain was used to capture live developmental apoptosis. Briefly, worms were propagated on a Nematode Growth Medium (NGM) plate seeded with OP50 (*Escherichia coli*). Ten microliters (10 μL) of M9 buffer were added to a coverslip. Early adult worms were picked and placed in the drop M9 buffer. Worms were cut at their midbodies by a needle to isolate embryos. Early-stage embryos were selected and placed on a poly-l-lysine coated slide. The slide was then mounted and sealed to avoid evaporation. A Leica Thunder Imager microscope, equipped with DIC condenser prisms, was used to capture 50 z stacks (1.83 μm each) every 90 s for 4 h, during which several apoptotic cells were detected. Representative images of a corresponding time, focused on an apoptotic cell, were selected for Figure 2 and the time-lapse movie (Video S1).

### 10.2. Electron Microscopy

Worms were irradiated with 120 grays; 24 h after irradiation, the germline was isolated by cutting *C. elegans* under the stereo microscope using 25× magnification. The isolated germline was fixed using 3.2% formaldehyde, 0.2% glutaraldehyde in 0.15 M sodium cacodylate buffer. The previously described procedure was followed [521]. Multiple overlapping electron micrographs were taken using a JEOL transmission electron microscope.

## Data Availability

The data that support the findings of this study are available from the corresponding author upon reasonable request.

## Supplementary Materials

The following are available online at https://www.mdpi.com/article/10.3390/ijms222212466/s1, Video S1: Developmental apoptosis during *C. elegans* embryogenesis.
